# Germline variants in DNA repair genes associated with hereditary breast and ovarian cancer syndrome: analysis of a 21 gene panel in the Brazilian population

**DOI:** 10.1186/s12920-019-0652-y

**Published:** 2020-02-10

**Authors:** Simone da Costa e Silva Carvalho, Nathalia Moreno Cury, Danielle Barbosa Brotto, Luiza Ferreira de Araujo, Reginaldo Cruz Alves Rosa, Lorena Alves Texeira, Jessica Rodrigues Plaça, Adriana Aparecida Marques, Kamila Chagas Peronni, Patricia de Cássia Ruy, Greice Andreotti Molfetta, Julio Cesar Moriguti, Dirce Maria Carraro, Edenir Inêz Palmero, Patricia Ashton-Prolla, Victor Evangelista de Faria Ferraz, Wilson Araujo Silva Jr

**Affiliations:** 1 Department of Genetics, Ribeirão Preto Medical School, University of São Paulo, Ribeirão Preto, SP Brazil; 2 Center for Medical Genomics at University Hospital of the Ribeirão Preto Medical School of University of São Paulo, Ribeirão Preto, SP Brazil; 3 Regional Blood Center at University Hospital of the Ribeirão Preto Medical School of University of São Paulo, Ribeirão Preto, SP Brazil; 4 Division of Internal Medicine and Geriatrics, Department of Internal Medicine, Ribeirão Preto Medical School, University of São Paulo, Ribeirão Preto, SP Brazil; 5 International Research, Center/CIPE, AC Camargo Cancer Center, Sao Paulo, SP Brazil; 6 Molecular Oncology Research Center, Barretos Cancer Hospital, Barretos, SP Brazil; 7 Laboratório de Medicina Genômica, Hospital de Clínicas de Porto Alegre, Porto Alegre, RS Brazil; 8 Department of Medical Genetics, University Hospital of the Ribeirão Preto Medical School, Ribeirão Preto, Brazil

**Keywords:** HBOC, DNA repair genes, Multi-gene panel screening, Next-generation sequencing, Molecular diagnosis, *BRCA1*, *BRCA2*

## Abstract

**Background:**

The Hereditary Breast and Ovarian Cancer Syndrome (HBOC) occurs in families with a history of breast/ovarian cancer, presenting an autosomal dominant inheritance pattern. *BRCA1* and *BRCA2* are high penetrance genes associated with an increased risk of up to 20-fold for breast and ovarian cancer. However, only 20–30% of HBOC cases present pathogenic variants in those genes, and other DNA repair genes have emerged as increasing the risk for HBOC. In Brazil, variants in *ATM, ATR, CHEK2, MLH1, MSH2, MSH6, POLQ, PTEN,* and *TP53* genes have been reported in up to 7.35% of the studied cases. Here we screened and characterized variants in 21 DNA repair genes in HBOC patients.

**Methods:**

We systematically analyzed 708 amplicons encompassing the coding and flanking regions of 21 genes related to DNA repair pathways (*ABRAXAS1*, *ATM, ATR, BARD1, BRCA1, BRCA2, BRIP1, CDH1, CHEK2, MLH1, MRE11, MSH2, MSH6, NBN, PALB2, PMS2, PTEN, RAD50, RAD51, TP53* and *UIMC1*). A total of 95 individuals with HBOC syndrome clinical suspicion in Southeast Brazil were sequenced, and 25 samples were evaluated for insertions/deletions in *BRCA1*/*BRCA2* genes. Identified variants were assessed in terms of population allele frequency and their functional effects were predicted through in silico algorithms.

**Results:**

We identified 80 variants in 19 genes. About 23.4% of the patients presented pathogenic variants in *BRCA1, BRCA2* and *TP53*, a frequency higher than that identified among previous studies in Brazil. We identified a novel variant in *ATR*, which was predicted as pathogenic by in silico tools. The association analysis revealed 13 missense variants in *ABRAXAS1, BARD1, BRCA2, CHEK2, CDH1, MLH1, PALB2,* and *PMS2* genes, as significantly associated with increased risk to HBOC, and the patients carrying those variants did not present large insertions or deletions in *BRCA1/BRCA2* genes.

**Conclusions:**

This study embodies the third report of a multi-gene analysis in the Brazilian population, and addresses the first report of many germline variants associated with HBOC in Brazil. Although further functional analyses are necessary to better characterize the contribution of those variants to the phenotype, these findings would improve the risk estimation and clinical follow-up of patients with HBOC clinical suspicion.

## Background

Hereditary Breast and Ovarian Cancer (HBOC) Syndrome occurs in families with a history of certain cancers, particularly breast and ovarian cancers with an autosomal dominant inheritance pattern. It encompasses about 5–10% of all breast cancer (BC) cases and up to 80% of all ovarian cancers (OC) [1, 2], and the affected families present a 50–80% increase in lifetime risk to BC and 30–50% to OC [3]. The National Comprehensive Cancer Network (NCCN) [4] is an alliance that creates the guidelines used for detection, prevention, as well as for adoption of strategies for risk reduction for HBOC affected families. According to NCCN, the main criteria used for further genetic risk evaluation in HBOC patients are: patients diagnosed with BC before 45 years or with invasive OC at any age, personal or familial recurrence of BC or OC, bilateral BC, and presence of male BC. Furthermore, patients at risk of HBOC may also present pancreatic and prostate cancers [4]. In this way, in order to help demystifying the association of HBOC with BC and OC risk in women [5], it has recently been proposed to change the name of HBOC to King Syndrome, in honor of Mary-Claire King who first described the locus associated with hereditary breast and ovarian cancers risk [6].

During the 1990’s, germline variants in the breast cancer susceptibility genes *BRCA1* and *BRCA2* were first described as showing increased risk for HBOC [7, 8]. Variants in *BRCA1* are associated with earlier-onset BC (30–50 years), when compared to *BRCA2* variants that increase the BC risk mainly for individuals of 40–60 years old [9]. The BC and OC risk rates also vary between *BRCA1* and *BRCA2* genes, with *BRCA1* carriers presenting a risk of up to 57% for BC and 40% for OC, while for *BRCA2* carriers the risk is slightly lower, 49 and 18% for BC and OC, respectively [10].

Molecular diagnosis is a very important step on the clinical management of HBOC patients since it allows for the family risk assessment, mortality reduction as well as allowing for the adoption of prophylactic measures, such as preventive mastectomy and/or oophorectomy, reducing the cancer risk by up to 95% in *BRCA1/BRCA2* carriers [11–13]. However, despite the high penetrance and the high frequency of variants found in *BRCA1/BRCA2* genes, only about 20% of hereditary BC and OC have been attributed to the presence of pathogenic variants in those genes, moreover, about 5–10% have been associated with other susceptibility genes, such as *TP53*, *STK11*, *PTEN*, *ATM,* and *CHEK2* [14]. Studies have demonstrated molecular diagnosis rates of about 4.6–54% when only *BRCA1/BRCA2* are screened, which evidences the association of other less penetrant genes with HBOC pathogenesis [15–18]. Even though the protocols for clinical management are well established for *BRCA1/BRCA2* carriers, patients tested negative for pathogenic *BRCA1/BRCA2* variants lack the proper clinical follow-up and genetic counselling when presenting similar clinical characteristics and BC/OC increased risk [19]. This reinforces the need of not only description but also the characterization of other genes associated with HBOC risk.

With the popularization of next-generation sequencing technologies (NGS), genes encoding proteins that work in the homologous recombination DNA repair pathway (HR), as well as mismatch repair (MMR) pathway, have been frequently reported as mutated in hereditary BC and OC cases [14, 16, 20–26]. Most genes are not only frequently mutated but they have also been considered by NCCN guidelines in the clinical management of patients at risk since they are associated with a high to moderate penetrance of BC and OC [4].

However, in the Brazilian population, besides *BRCA1* and *BRCA2*, the characterization of other DNA repair genes related to HBOC susceptibility is still in its infancy. The main available data encompasses the screening of hotspot variants and microdeletions in *CHEK2*, *PTEN*, *POLQ* and *TP53* genes [2, 27–30], and to date, only two studies using NGS technology are available in Brazil. Recently, the screening of the whole exome in Brazilian patients negative for *BRCA1/BRCA2* pathogenic variants revealed other genes, such as *ATM* and *BARD1,* carrying pathogenic variants [26]. Another study using multi-gene screening showed a prevalence of 9.8% of patients carrying *BRCA1/BRCA2* pathogenic variants and 4.5% carrying pathogenic variants in *ATR, CDH1, MLH1* and *MSH6* genes [24].

In this study, we screened 95 samples of patients with HBOC syndrome clinical suspicion, using a multi-gene panel sequencing both flanking and coding regions of *BRCA1, BRCA2* and another 19 DNA repair genes. Also, 25 samples were tested for *BRCA1/BRCA2* copy number variations (CNVs). The molecular screening was performed to identify causal germline variants and characterize variants of unknown/uncertain significance (VUS) in order to improve the molecular diagnosis. Our data report a global analysis of 21 DNA repair genes to the HBOC etiology, which are contributing to the epidemiology of HBOC in Brazil.

## Methods

### Patient samples and clinical data

The individuals evaluated were referred to the Cancer Genetics Counseling Service of the University Hospital of the Ribeirão Preto Medical School of the University of São Paulo (HCFMRP-USP, Ribeirão Preto – Brazil) for cancer risk assessment from 2008 to 2016. A total of 95 unrelated subjects were eligible for further investigation. These individuals had a clinical suspicion of HBOC Syndrome, and presented criteria for genetic risk evaluation according to the NCCN Clinical Practice Guidelines in Oncology v.2.2015 [4], and presented a cumulative risk of *BRCA1* and *BRCA2* variants higher than 10%, using PennII model (https://pennmodel2.pmacs.upenn.edu/penn2/), and a personal history of cancer.

The clinical and pathologic data was abstracted from medical records of the HCFMRP-USP and included personal and family cancer histories, cancer histology, stage, and receptor status. The College of American Pathologists (CAP) guidelines were used to define progesterone receptor (PR) and human epidermal growth factor receptor 2 (HER2) positivity, but for estrogen receptors we used the 10% threshold for positivity [31].

Samples of 28 elderly people (over 70 years old) negative for personal history of cancer, were used as control group and had their whole exome sequenced by the Molecular Genetics Laboratory of UNICAMP (Campinas, SP), headed by Dr. Iscia Lopes Cendes, who kindly provided the results. We believe that older people with no personal cancer history constitute a proper control for hereditary cancer studies once those people over the age of developing hereditary cancer and reached old age free of this disease. Therefore, if any variants are found in both HBOC and elderly cohorts, we discourage further associations with breast and ovary cancer risk.

Genomic DNA of both HBOC and elderly cohorts were extracted from whole blood using the Wizard® Genomic DNA Purification Kit (Promega, Madison, WI). The samples were part of the Center for Medical Genomics Biorepository (HCFMRP-USP) and were used for these analyses only after approval by the Ethics Research Committee of the HCFMRP-USP (n. 2819/2016).

The genetic test results from this analysis were returned to study participants, helping the clinical decision when suitable.

### Multi-gene panel screening

We used a TruSeq Custom Amplicon Library Preparation Kit (Illumina, San Diego, CA) for the enrichment of coding and flaking regions of 21 DNA repair genes (*ABRAXAS1, ATM, ATR, BARD1, BRCA1, BRCA2, BRIP1, CDH1, CHEK2, MLH1, MRE11, MSH2, MSH6, NBN, PALB2, PMS2, PTEN, RAD50, RAD51, TP53* and *UIMC1*). A total of 708 amplicons for a 98% mean coverage were custom designed using the Illumina Design Studio (Illumina, San Diego, CA). Paired-end sequencing was performed on MiSeq equipment (Illumina, San Diego, CA), using the MiSeq sequencing kit v2 (2 × 250) (Illumina, San Diego, CA). The base call files (bcl) files were converted into fastq using the FASTQ Generation v.1.0.0 software, available on BaseSpace (Illumina, San Diego, CA). The mapping and variant calling were performed using Burrows-Wheeler Alignment (BWA) mem tool, and Haplotype Caller, respectively, following the GATK v.3.6–0 (https://software.broadinstitute.org/gatk/) best practices guidelines for germline single nucleotide polymorphisms (SNPs) and insertion/deletions (indels) detection, using the GRCh37.75/hg19 as reference genome (http://hgdownload.cse.ucsc.edu/). We used Snpeff for variant annotation (http://snpeff.sourceforge.net/).

The graphics to represent the sequencing data were built using the Bioconductor (https://www.bioconductor.org/) GenVisR [32] and ComplexHeatmap [33] packages on R environment (RStudio, version 1.2.1335).

### Variants classification and prioritization

All variants were classified according to recommendations of the American College of Medical Genetics and Genomics and the Association for Molecular Pathology (ACMG/AMP) consensus [34] using the VarSome variant search engine [35]. For a more accurate variant characterization, we also assessed the ClinVar classification (https://www.ncbi.nlm.nih.gov/clinvar/), and the pathogenicity scores of the 6 following in silico prediction tools: CADD [36], AlignGVGD [37], UMD-Predictor [38], SIFT [39], Poly-Phen [40] and MutationTaster [41].

In order to prioritize a smaller number of variants for further characterization, we refined the whole set of variants in favor of remaining with those classified as pathogenic according to ACMG/AMP consensus, as well as remaining with all the VUS and benign variants (according to VarSome and ClinVar) which presented both in coding and splicing regions, if they were predicted as damaging/pathogenic by the in silico prediction tools. We decided to maintain the benign variants in this set of prioritized variants in order to avoid disregarding variants of potential effect to the phenotype, since ClinVar and VarSome classifications are not always supported by strong evidences (segregational and functional data). Thereafter, at times we refer to those variants as presenting conflicting data on pathogenicity.

### Sanger Sequencing Validation

All samples that presented pathogenic variants, as well as all those significantly associated with relative risk to HBOC were submitted to Sanger sequencing. Briefly, 100 ng of whole blood DNA from individuals carrying those variants was submitted to PCR amplification performed with Taq DNA polymerase (Promega, Madison, WI). The amplification products were sequenced in both directions using BigDye Terminator v3.1 (Life Technologies, Carlsbad, CA) and specific primers for each region, in the ABI 3500XL Genetic Analyzer (Life Technologies, Carlsbad, CA), according to manufacturer’s instructions. Sequencing data were analyzed with the Geneious R7 software v7.1 using the GRCh37/hg19 sequence as reference. Primer sequences are available under request.

### Analysis of CNVs in *BRCA1* and *BRCA2* genes

To exclude the presence of large insertions/deletions in *BRCA1/BRCA2* genes that might not have been detected by NGS, we performed the Multiplex Ligation-dependent Probe Amplification (MLPA) analysis for patients who did not present any variants on *BRCA1/BRCA2* (*n* = 12) after the multi-gene panel screening, as well as for those patients carrying variants that were significantly associated with relative risk to HBOC (*n* = 15). In order to achieve this, we used the P087-BRCA1 and P090-BRCA2 kits (MRC-Holand, Amsterdam, NH), according to the manufacturer’s recommendations. Briefly, the DNA from HBOC patients and control samples were pre-heated to 98 °C, and then the salt solution and probe mix were added to the DNA. After the ligation of annealed nucleotides, the targeted genes were amplified using polymerase chain reaction (PCR). PCR products were separated using the ABI3500XL Genetic Analyzer (Applied Biosystems, Foster City, CA), and the fragments were analysed using the Coffalyser software v.140701.0000 (MRC-Holand, Amsterdam, NH).

### Screening for the c.156_157insAlu variant in *BRCA2*

All 95 HBOC samples were screened for the variant c.156_157insAlu in the *BRCA2* gene, which was not detected by the multi-gene panel analysis. We performed two rounds of PCR: a first PCR reaction for *BRCA2* exon 3 amplification (forward primer: GTCACTGGTTAAAACTAAGGTGGGA and reverse primer: GAAGCCAGCTGATTATAAGATGGTT), and a second PCR specific for Alu fragment amplification (forward primer: GACACCATCCCGGCTGAAA, reverse primer: CCCCAGTCTACCATATTGCAT). The cycling conditions were 94 °C for 3 min, 35 cycles at 94 °C for 1 min, 52 °C for 1 min, and 72 °C for 4 min, and a final extension of 72 °C for 10 min. For the sample that presented a fragment amplification bigger than that expected for *BRCA2* exon 3 amplification (around 200pb), the specific Alu PCR was performed using the same cycling conditions applied for *BRCA2* exon 3 amplification. The PCR product was then sequenced in both directions using BigDye Terminator v3.1 (Life Technologies, Carlsbad, CA,) and Alu specific primers in the ABI 3500XL Genetic Analyzer (Life Technologies, Carlsbad, CA), according to manufacturer’s instructions.

### Haplotype analysis for high frequency *BRCA1* benign variants

We performed a haplotype analysis in order to assess if five high frequency *BRCA1* variants (c.*421G > T, p.Pro871Leu, p.Glu1038Gly, p.Lys1183Arg, and p.Ser1613Gly) were segregating together and were associated with HBOC risk. Based on previous results of our group, which also found these *BRCA1* variants presenting a high frequency in a small HBOC cohort (*n* = 25, unpublished data), we joined the two HBOC cohorts (*n* = 94 sequenced in this study, and n = 25 samples previously screened for those variants, totalizing a final *n* = 119) and also genotyped 108 additional elderly samples for the five *BRCA1* SNVs (*n* = 28 sequenced in this study, and *n* = 108 additional elderly samples, totalizing a final *n* = 136) to perform a more accurate statistical analysis.

Additionally, in order to assess the frequency of those five *BRCA1* SNVs in other Brazilian populations, we genotyped 94 HBOC versus 94 control samples from Porto Alegre Clinical Hospital (Porto Alegre, RS, Brazil); 171 HBOC versus 185 control samples from A.C. Camargo Cancer Center (São Paulo, SP, Brazil), and also 72 HBOC versus 72 control samples from Barretos Cancer Hospital (Barretos, SP, Brazil). We then performed the haplotype analysis.

We applied a TaqMan Allele Discrimination assay (Applied Biosystems, Foster City, CA), using designed probes and primers specific to each *BRCA1* variant: c.*421G > T (assay ID: AHX1AK8), p.Pro871Leu (assay ID: C___2287943_10), p.Glu1038Gly (assay ID: C_2287888_10), p.Lys1183Arg (C___2287889_20), and p.Ser1613Gly (assay ID: C_2615208_20). For each reaction, we used 2 μL of each sample at 5 ng/μL, 5 μL of TaqMan master mix (Applied Biosystems, Foster City, CA), and 0.25 μL (200 nM) of each probe, reaching a final volume of 10 μL, placed in 96-well PCR plates. The cycling conditions were 95 °C for 10 min, 40 cycles at 92 °C for 15 s and 60 °C for 1 min, and 60 °C for 1 min, and a final extension at 72 °C for 10 min. The amplification was performed using the 7500 Real-Time PCR Systems (Applied Biosystems, Foster City, CA) and the results were analysed using the manufacturer’s software.

Subsequently, we assessed the haplotype frequency estimation for all samples using the haplo.stats package version 1.7.9 (https://cran.r-project.org/web/packages/haplo.stats/index.html), on R environment (RStudio, version 1.2.1335). The haplo.stats analysis also estimates the association among haplotypes and the disease, considering *p* value <0.05 as statically significant.

### Risk association analysis and statistical tests

For the risk association analysis we used the allele frequencies found in our HBOC cohort, compared to the allele frequencies of the same variants available in the AbraOM public database which includes the exome sequencing data of 609 elderly Brazilians [42]. We decided to use public databases instead of the allele frequencies on the elderly samples due to low number of individuals sequenced. When the allele frequencies on AbraOM were zero, we used the European non-Finnish, Latin, American, African and frequencies available on 1000 Genomes [43] or ExAC [44] databases. We performed an odds ratio (OR) analysis applying the Fisher’s exact test. The *p*-values were assessed using the Pearson’s X^2^ test.

For assessing the clinical and molecular associations, we applied Pearson’s X^2^ test.

For these two analyses we used the R commander [45] tools on R environment (RStudio, version 1.2.1335) and considered results as statistically significant at a p-value of 0.05 or less.

For the survival (Kaplan Meier) analysis, we used Logrank test for trend and Mantel-Cox, as recommended by GraphPad Prism 8.1.2. We also assessed the results for the Gehan-Breslow-Wilcoxon test.

## Results

### Patients clinical characterization

Most of patients (*n* = 84) were diagnosed with breast cancer, showing a prevalence of 82.4% (*n* = 80) of Invasive Ductal Carcinoma (IDC) (Additional file 1: Table S1). The Luminal and Triple-negative (TN) were the most frequent molecular subtypes, presenting a frequency of 33.3 and 28.6% of BC cases, respectively. In general, most of the patients (*n* = 65) presented tumors of intermediate to high grades (2 and 3), independently to the age of diagnosis. Only six patients (6.3%) were diagnosed with ovarian cancer, of which half of cases were serous ovarian cancer (Table 1, and Additional files 1: Table S1). One patient presented with diffuse gastric cancer (the only man in our cohort) and another, endometrial adenocarcinoma, and both presented with a strong history of breast and ovarian cancers in their families. Only one case presented with both asynchronous BC and OC. Most of the cases (85.3%) were diagnosed between 22 and 49 years, and 13.6% (*n* = 13) deceased due to distant metastasis occurrence (Table 1).

**Table 1:** Phenotypic and genotypic characterization of the HBOC cohort according to BRCA mutational status

Variable	Mutational status						*p*-value^&^
	BRCA pathogenic^a^		BRCA Benign and VUS^b^		non-BRCA		
	*n* = 17	%	*n* = 65	%	*n* = 12	%	
**Gender**							
Man			1	1.5			
Woman	17	18.1	64	98.5	12	100	
**Age at diagnosis (median)**	24–57 (34)		22–72 (37)		31–47 (36.5)		
**Deaths**	1	5.9	11	16.9	2	16.6	0.0927
**Survival in years (median)**	8		3		8		
**Familial history**							
Present	14	82.3	52	80	10	83.3	0.294
Absent	3	17.7	11	16.9	2	16.7	
NI			2	3.1			
**Tumor site**							
Breast	17	100	57	87.7	12	100	0.6034
Ovary			6	9.3			
Edometrium			1	1.5			
Stomach			1	1.5			
**Tumor distribution**							
Unilateral or located	12	70.6	48	73.8	10	83.3	0.2376
Bilateral (breast)	5	29.4	6	9.3	1	8.3	
Multiple tumors			5	7.7			
NI			6	9.3	1	8.3	
**Breast molecular subype**							
Luminal	4	23.5	20	30.8	5	41.7	0.4425
Luminal HER	2	11.8	11	16.9	3	25	
HER2	2	11.8	7	10.8	1	8.3	
TN	9	52.9	13	20	1	8.3	
PR			1	1.5			
NI			13	20	2	16.7	
**Tumor grade**							
1	1	5.9	7	10.8	1	8.3	0.03686
2	3	17.6	29	44.6	5	41.7	
3	11	64.7	11	16.9	4	33.3	
NI	2	11.8	18	27.7	2	16.7	
**Lymph node metastasis**							
Present	7	41.2	31	47.7	7	58.3	0.1984
Absent	8	47.1	16	24.6	3	25	
NI	2	11.8	18	27.7	2	16.7	
**Distant metastasis**							
M0	1	5.9	38	58.5	7	58.3	0.1964
M1	15	88.2	14	21.5	3	25	
NI	1	5.9	13	20	2	16.7	

^a^Variants previously characterized as pathogenic (ClinVar). ^b^Patients carrying benign or variants of unknown significance on *BRCA1/BRCA2* genes. ^&^The association between the genotypes and the clinical characteristics were calculated using the Pearson’s X^2^ test. *HER2* When the HER2 protein is overexpressed; *TN* Triple-negative, *PR* Positive for progesterone receptors, *NI* Not-informed

### Multi-gene panel screening

We identified 667 single nucleotide variants (SNVs) and small insertions/deletions in 94 out of 95 samples screened for variants in their coding and flanking regions of 21 DNA repair genes. One sample was excluded due to a general low quality in the base calling. We then prioritized variants filtering it according to the following criteria: 1 – Variants classified as pathogenic according to ACMG/AMP consensus, and 2 - VUS and benign variants present both in coding and splicing regions, and predicted as damaging/pathogenic by the in silico prediction tools. This filtering aimed to select the possible candidate variants without losing variants of unknown significance (VUS), which were not yet characterized but may exert some effect to the phenotype. We selected 82 variants in 19 genes with *RAD50* and *PTEN* presenting no possible candidate variants (Table 2). Considering these prioritized variants, about 81% of the patients presented variants in *BRCA1* gene, although genes such as *ABRAXAS1, ATM*, *BRCA2* and *UIMC1* also emerged as presenting a high frequency of variants in our cohort*.* Only 3% of the prioritized variants are described in the breast (*TP53* and *MLH1* variants) and ovarian cancer (*BRCA2* variant) samples of The Cancer Genome Atlas database (TCGA) (https://www.cbioportal.org/), which is expected once the publicly available data on TCGA comprises solely somatic variants.

**Table 2:** Prioritized variants identified in the HBOC cohort and its pathogenicity prediction

Gene	Variant nomenclature		dbSNP ID	Variant type	Varsome	ClinVar	In silico Predictions						Sample ID
	Coding DNA	Protein					CADD	AlignGVGD	UMD PREDICTOR	SIFT	PolyPhen	Mutation Taster	
**HR genes**													
*ATM*	c.1541G > A	p.Gly514Asp	rs2235000	missense	Benign	Benign/Likely Benign	25.7	Class C65	Polymorphism	Tolerated	Probably damaging	Disease Causing	3664; 4146
	c.1636C > G	p.Leu546Val	rs2227924	missense	Likely Benign	Benign/Likely Benign	11.58	Class C25	Polymorphism	Damaging	Possibly damaging	Polymorphism	3617; 3634
	c.1810C > T	p.Pro604Ser	rs2227922	missense	Uncertain Significance	Benign/Likely Benign	23.3	Class C65	Probably polymorphism	Tolerated	Possibly damaging	Disease Causing	2775
	c.2442C > A	p.Asp814Glu	rs3218695	missense	Likely Benign	Benign	15.88	Class C35	Polymorphism	Tolerated	Benign	Polymorphism	2753; 2784
	c.2572 T > C	p.Phe858Leu	rs1800056	missense	Uncertain Significance	Conflicting interpretations of pathogenicity	13.50	Class C15	Polymorphism	Damaging	Possibly damaging	Polymorphism	4268
	c.4258C > T	p.Leu1420Phe	rs1800058	missense	Uncertain Significance	Conflicting interpretations of pathogenicity	15.47	Class C15	Polymorphism	Tolerated	Benign	Disease Causing	3650
	c.5557G > A	p.Asp1853Asn	rs1801516	missense	Benign	Benign/Likely Benign	23.2	Class C15	Polymorphism	Tolerated	Benign	Polymorphism	2699; 2724; 2775; 3002; 3132 (homoz); 3141; 3166; 3187; 3728 (homoz); 4063; 4133; 4135; 4137; 4138 (homoz); 4147; 4226 (homoz)
	c.6995 T > C	p.Leu2332Pro	rs4988111	missense	Likely Benign	Benign/Likely Benign	15.87	Class C65	Polymorphism	Tolerated	Benign	Polymorphism	3617; 3634
	c.7740A > C	p.Arg2580Ser	rs199915459	missense	Uncertain Significance	Uncertain significance	15.65	Class C65	Pathogenic	Tolerated	Benign	Disease Causing	3671
	c.5558A > T	p.Asp1853Val	rs1801673	missense	Uncertain Significance	Conflicting interpretations of pathogenicity	24.2	Class C65	Pathogenic	Damaging	Possibly damaging	Disease Causing	4186; 4264
*ATR*	c.2794C > A	p.Pro932Thr	–	missense	Uncertain Significance	–	27.0	Class C35	Pathogenic	Damaging	Probably damaging	Disease Causing	4020
	c.7300C > G	p.Pro2434Ala	rs33972295	missense	Likely Benign	Benign/Likely Benign	23.5	Class C25	Polymorphism	Damaging	Probably damaging	Disease Causing	4228
	c.946G > A	p.Val316Ile	rs28897764	missense	Likely Benign	Benign/Likely Benign	18.61	Class C25	Probable polymorphism	Tolerated	Benign	Disease Causing	2726; 3116; 3671; 3703; 4228
*BARD1*	c.-83C > T	–	rs71579840	5’UTR premature start codon gain	Likely Benign	–	8485	–	–	–	–	–	3002
	c.1972C > T	p.Arg658Cys	rs3738888	missense	Uncertain Significance	Benign/Likely Benign	26.5	Class C65	Probably pathogenic	Damaging	Probably damaging	Disease Causing	3671
	c.1268A > G	p.Lys423Arg	rs749383704	missense	Uncertain Significance	Uncertain significance	21.8	Class C25	Probably pathogenic	Tolerated	Benign	Disease Causing	2995
	c.764A > G	p.Asn255Ser	rs138904906	missense	Likely Benign	Uncertain significance	16.75	Class C45	Polymorphism	Tolerated	Probably damaging	Polymorphism	3716
	c.716 T > A	p.Leu239Gln	rs200359745	missense	Likely Benign	Uncertain significance	1061	Class C65	Probably pathogenic	Tolerated	Benign	Polymorphism	3051
*BRCA1*	c.*421G > T	–	rs8176318	3’UTR	Benign	Benign	4.78	–	–	–	–	–	2697 (homoz); 2699; 2742; 2750; 2753; 2779; 2801; 2815; 2972; 2977; 3002; 3056; 3078; 3097; 3114; 3115; 3116; 3132; 3141; 3166; 3227; 3462; 3617; 3671; 3703; 3728; 3806; 3842; 3897; 4016; 4020; 4135; 4138; 4146; 4147; 4161; 4166; 4177; 4220; 4226; 4228; 4279
	c.3119G > A	p.Ser1040Asn	rs4986852	missense	Likely Benign	Benign	14.86	Class C45	Polymorphism	Tolerated	Probably damaging	Polymorphism	2699; 2785; 2995; 3002; 3114; 3876; 4020; 4037; 4132
	c.5019G > A	p.Met1673Ile	rs1799967	missense	Benign	Benign	22.0	Class C0	Probable polymorphism	Tolerated	Benign	Disease Causing	3897
	c.4598G > T	p.Ser1533Ile	rs1800744	missense	Likely Benign	Benign	16.14	Class C65	Polymorphism	Damaging	Possibly damaging	Polymorphism	3113
	c.1648A > C	p.Asn550His	rs56012641	missense	Likely Benign	Benign	17.67	Class C65	Polymorphism	Damaging	Probably damaging	Polymorphism	4132
	c.1067A > G	p.Gln356Arg	rs1799950	missense	Benign	Benign	17.80	Class C35	Polymorphism	Damaging	Probably damaging	Polymorphism	2724; 2775; 3187; 3703; 4133; 4139
	c.2077G > A	p.Asp693Asn	rs4986850	missense	Benign	Benign	15.84	Class C15	Polymorphism	Damaging	Benign	Polymorphism	2815; 2977; 3097; 3116; 3671; 4122; 4220
	c.5507G > A	p.Trp1836Ter	rs80356962	stop gained	Pathogenic	Pathogenic	44	–	Pathogenic	–	–	Disease Causing	3051
	c.5329dupC	p.Gln1756Profs*74	rs397507247	frameshift	Pathogenic	Pathogenic	35	–	–	–	–	–	2812; 3132; 3141; 3155; 3639; 3722; 3728; 4093; 4135; 4137; 4186
	c.3331_3334delCAAG	p.Gln1111Asnfs*5	rs80357701	frameshift	Pathogenic	Pathogenic	23.7	–	–	–	–	–	2723
	c.2612C > T	p.Pro871Leu	rs799917	missense	Benign	Benign	17.97	Class C65	–	Tolerated	Benign	Polymorphism	2697 (homoz); 2699; 2724; 2726; 2742 (homoz); 2750; 2753; 2779 (homoz); 2801; 2812; 2815; 2972; 2977; 3002; 3056 (homoz); 3078 (homoz); 3083; 3097; 3114; 3115 (homoz); 3116 (homoz); 3132; 3141; 3166; 3227; 3441(homoz); 3462; 3617(homoz); 3650(homoz); 3651; 3664(homoz); 3671; 3703; 3728; 3782; 3802; 3806; 3842(homoz); 3897; 3920; 4016; 4020; 4037; 4063; 4093; 4122; 4135; 4138; 4139; 4144; 4146(homoz); 4147; 4161; 4166 (homoz); 4177; 4186 (homoz); 4214; 4220; 4226 (homoz); 4228; 4250 (homoz); 4262; 4268 (homoz); 4279
	c.3548A > G	p.Lys1183Arg	rs16942	missense	Benign	Benign		Class C25	–	Tolerated	Benign	Polymorphism	2697 (homoz); 2699; 2742; 2750; 2753; 2801; 2815; 2972; 2977; 3002; 3056; 3078 (homoz); 3097; 3114; 3116; 3141; 3166; 3462; 3617 (homoz); 3651; 3703; 3806; 3842 (homoz); 3897; 4016; 4020; 4122; 4135; 4138; 4146; 4161; 4166; 4262; 4268 (homoz); 4279
	c.4900A > G	p.Ser1613Gly	rs1799966	missense	Benign	Benign		Class C55	Polymorphism	Damaging	Possibly damaging	Polymorphism	2697; 2699; 2742; 2750; 2753; 2779; 2784; 2801; 2815; 2972; 2977; 3002; 3056; 3078 (homoz); 3097; 3114; 3115; 3116; 3132; 3141; 3166; 3227; 3462; 3617 (homoz); 3651; 3671; 3703; 3728; 3782; 3806; 3842 (homoz); 3897; 4016; 4020; 4122; 4135; 4138; 4146; 4147; 4161; 4166; 4177; 4220; 4226; 4228; 4262; 4268 (homoz); 4279
	c.536A > G	p.Tyr179Cys	rs56187033	missense	Likely Benign	Benign	24.7	Class C65	Pathogenic	Damaging	Probably damaging	Disease Causing	4132
	c.591C > T	p.Cys197Cys	rs1799965	splice region	Likely Benign	Uncertain significance	14.63	–	Probably pathogenic	–	–	–	4063
	c.3113A > G	p.Glu1038Gly	rs16941	missense	Benign	Benign	22.2	Class C65	Polymorphism	Damaging	Possibly damaging	Polymorphism	2697; 2699; 2742; 2750; 2753; 2779; 2784; 2801; 2815; 2972; 2977; 3002; 3056; 3078 (homoz); 3097; 3114; 3115; 3116; 3132; 3141; 3166; 3227; 3462; 3617 (homoz); 3651; 3671; 3703; 3728; 3782; 3806; 3842; 3897; 4016; 4020; 4122; 4135; 4138; 4146; 4147; 4161; 4166; 4220; 4226; 4228; 4262; 4268 (homoz); 4279
*BRCA2*	c.156_157insAlu	–	–	insertion	Pathogenic	Pathogenic	–	–	–	–	–	–	
	c.811G > A	p.Gly271Arg	rs786204274	missense	Uncertain Significance	Conflicting interpretations of pathogenicity	20.6	Class C65	Probably pathogenic	Damaging	Benign	Polymorphism	2977
	c.3869G > A	p.Cys1290Tyr	rs41293485	missense	Likely Benign	Benign	14.39	Class C65	Probably pathogenic	Tolerated	Benign	Polymorphism	3662
	c.4258G > T	p.Asp1420Tyr	rs28897727	missense	Likely Benign	Benign	15.81	Class C65	Polymorphism	Damaging	Benign	Polymorphism	3649
	c.6100C > T	p.Arg2034Cys	rs1799954	missense	Likely Benign	Benign	20.4	Class C65	Polymorphism	Damaging	Possibly damaging	Polymorphism	3441; 3617; 4279
	c.8149G > T	p.Ala2717Ser	rs28897747	missense	Likely Benign	Benign	15.38	Class C65	Polymorphism	Tolerated	Possibly damaging	Polymorphism	3002
	c.8850G > T	p.Lys2950Asn	rs28897754	missense	Uncertain Significance	Conflicting interpretations of pathogenicity	22.4	Class C65	Probable polymorphism	Damaging	Probably damaging	Disease Causing	2781
	c.865A > C	p.Asn289His	rs766173	missense	Benign	Benign	17.23	Class C65	Polymorphism	Damaging	Probably damaging	Polymorphism	2995; 3051; 3056; 3651; 3722; 4166
	c.2808_2811delACAA	p.Ala938Profs*21	rs80359351	frameshift	Pathogenic	Pathogenic	24.3	–	–	–	–	–	4147
	c.8851G > A	p.Ala2951Thr	rs11571769	missense	Likely Benign	Benign	26.2	Class C55	Polymorphism	Damaging	Probably damaging	Disease Causing	3051
	c.9026_9030delATCAT	p.Tyr3009Serfs*7	rs80359741	frameshift	Pathogenic	Pathogenic		–	–	–	–	–	2785
	c.9382C > T	p.Arg3128Ter	rs80359212	stop gained	Pathogenic	Pathogenic	48	–	Pathogenic	–	–	Disease Causing	4262
	c.9976A > T	p.Lys3326Ter	rs11571833	stop gained	Likely Benign	Benign	36	–	Pathogenic	–	–	Disease Causing	3650
	c.8324 T > G	p.Met2775Arg	rs80359073	missense	Uncertain Significance	Conflicting interpretations of pathogenicity	24.1	Class C65	Pathogenic	Tolerated	Benign	Disease Causing	3116
*BRIP1*	c.3693A > G	p.Ile1231Met	rs876659290	missense	Uncertain Significance	Uncertain significance	13.29	Class C0	Polymorphism	Damaging	Benign	Polymorphism	3132; 3728
	c.517C > T	p.Arg173Cys	rs4988345	missense	Likely Benign	Benign/Likely Benign	24.9	Class C65	Pathogenic	Damaging	Probably damaging	Disease Causing	4122; 4173
	c.2220G > T	p.Gln740His	rs45589637	missense	Likely Benign	Uncertain significance	12.53	Class C15	Probably pathogenic	Damaging	Probably damaging	Disease Causing	3078
*CHEK2*	c.410G > A	p.Arg137Gln	rs368570187	missense	Likely Benign	Likely Benign	16.54	Class C35	Probable polymorphism	Tolerated	Benign	Disease Causing	3116
	c.480A > G	p.Ile160Met	rs575910805	missense	Uncertain Significance	Conflicting interpretations of pathogenicity	22.6	Class C0	Probable polymorphism	Damaging	Probably damaging	Disease Causing	3097
*FAM175A/ABRAXAS1*	c.489G > T	p.Arg163Ser	rs535462791	missense	Uncertain Significance	–	22.3	Class C65	Pathogenic	Tolerated	Possibly damaging	Disease Causing	3187
	c.1042G > A	p.Ala348Thr	rs12642536	missense	Benign	–	13.44	Class C55	Polymorphism	Damaging	Possibly damaging	Polymorphism	2697; 2699; 2723; 2724; 2726; 2742; 2750; 2754 (homoz); 2775; 2779; 2781 (homoz); 2784 (homoz); 2812; 2972; 2977; 3002; 3078; 3097; 3113 (homoz); 3114 (homoz); 3115; 3132 (homoz); 3141 (homoz), 3155 (homoz); 3166 (homoz); 3441; 3462; 3617; 3634; 3639; 3649 (homoz); 3651; 3662; 3706; 3716; 3722; 3728 (homoz); 3761; 3772; 3806; 3876; 3897; 3920; 4016; 4037; 4063; 4093; 4132 (homoz); 4137; 4144; 4145; 4147; 4161; 4166; 4177; 4226 (homoz); 4228; 4250; 4259; 4264; 4279
*MRE11*	c.1011C > G	p.Ser337Arg	rs115244417	missense	Likely Benign	Benign	21.9	Class C65	Probable polymorphism	Tolerated	Benign	Disease Causing	4135
	c.2101A > G	p.Met701Val	rs1805362	missense	Likely Benign	Benign/Likely Benign	16.49	Class C15	Polymorphism	Damaging	Benign	Disease Causing	3650
*NBN*	c.202 T > G	p.Leu68Val	rs1200599843	missense	Uncertain Significance	Uncertain significance	15.95	Class C25	Polymorphism	Tolerated	Benign	Disease Causing	2785
	c.797C > T	p.Pro266Leu	rs769420	missense	Likely Benign	Benign	24.6	Class C65	Polymorphism	Damaging	Probably damaging	Disease Causing	3078
*PALB2*	c.2794G > A	p.Val932Met	rs45624036	missense	Likely Benign	Benign/Likely Benign	25.3	Class C15	Polymorphism	Tolerated	Probably damaging	Disease Causing	3842
	c.53A > G	p.Lys18Arg	rs138789658	missense	Uncertain Significance	Conflicting interpretations of pathogenicity	24.3	Class C25	Polymorphism	Damaging	Probably damaging	Polymorphism	3897; 4037
	c.949A > C	p.Thr317Pro	rs587780223	missense	Likely Benign	Uncertain significance	4.012	Class C35	Polymorphism	Tolerated	Possibly damaging	Polymorphism	4139
*RAD51*	c.164C > T	p.Ala55Val	rs145617142	missense	Uncertain Significance	Uncertain significance	23.6	Class C55	Probable polymorphism	Tolerated	Possibly damaging	Disease Causing	3116
*UIMC1*	c.43C > T	p.Arg15Trp	rs13167812	missense	Uncertain Significance	–	22.5	Class C65	Polymorphism	Damaging	Probably damaging	Polymorphism	2750; 4250
	c.1304C > T	p.Pro435Leu	rs3733876	missense	Benign	–	23.8	Class C65	Polymorphism	Damaging	Probably damaging	Polymorphism	2699; 2724; 2754; 2815; 3056; 3083; 3132 (homoz); 3634; 3649; 3671; 3761; 3842; 3920; 4020; 4063 (homoz); 4138; 4173; 4214
**MMR genes**													
*MLH1*	c.306G > A	p.Glu102Glu	rs63751665	splice region	Likely Benign	uncertain significance	22.4	–	Pathogenic	–	–	Disease Causing	4020
	c.637G > A	p.Val213Met	rs2308317	missense	Likely Benign	Benign	23.9	Class C15	Polymorphism	Tolerated	Possibly damaging	Disease Causing	4020
	c.1217G > A	p.Ser406Asn	rs41294980	missense	Likely Benign	Benign	13.09	Class C45	Polymorphism	Tolerated	Possibly damaging	Polymorphism	2963
	c.2146G > A	p.Val716Met	rs35831931	missense	Likely Benign	Benign	24.4	Class C15	Probable polymorphism	Damaging	Probably damaging	Disease Causing	2754
*MSH2*	c.2500G > A	p.Ala834Thr	rs63750757	missense	Likely Benign	Likely Benign	33.00	Class C55	Pathogenic	Damaging	Probably damaging	Disease Causing	4214
	c.380A > G	p.Asn127Ser	rs17217772	missense	Benign	Benign	22.7	Class C45	Probable polymorphism	Damaging	Possibly damaging	Disease Causing	3650; 3920; 4146; 4228
	c.965G > A	p.Gly322Asp	rs4987188	missense	Likely Benign	Benign	23.0	Class C65	Probable polymorphism	Tolerated	Possibly damaging	Disease Causing	2815; 3113; 3441; 4264
*MSH6*	c.1186C > G	p.Leu396Val	rs2020908	missense	Likely Benign	Benign	16.97	Class C25	Polymorphism	Tolerated	Possibly damaging	Disease Causing	2699; 2754
	c.2633 T > C	p.Val878Ala	rs2020912	missense	Likely Benign	Benign	10.23	Class C55	Polymorphism	Tolerated	Benign	Disease Causing	4147
*PMS2*	c.59G > A	p.Arg20Gln	rs10254120	missense	Benign	Benign	16.65	Class C35	Polymorphism	Tolerated	Possibly damaging	Polymorphism	4146; 2963; 3097; 3116; 3722; 3782; 3806; 4137; 4145; 4220; 4262
	c.2374G > A	p.Asp792Asn	rs587781265	missense	Uncertain Significance	Uncertain significance	29.8	Class C15	Probably pathogenic	Damaging	Probably damaging	Disease Causing	3802
	c.2350G > A	p.Asp784Asn	rs143340522	missense	Uncertain Significance	Uncertain significance	27.8	Class C15	Polymorphism	Damaging	Probably damaging	Disease Causing	4264
	c.2149G > A	p.Val717Met	rs201671325	missense	Uncertain Significance	Conflicting interpretations of pathogenicity	20.7	Class C15	Probable polymorphism	Damaging	Probably damaging	Disease Causing	3116; 3462
	c.1866G > A	p.Met622Ile	rs1805324	missense	Likely Benign	Benign	18.96	Class C0	Polymorphism	Tolerated	Possibly damaging	Disease Causing	3772
	c.1688G > T	p.Arg563Leu	rs63750668	missense	Likely Benign	Benign/Likely Benign	10.65	Class C65	Polymorphism	Tolerated	Possibly damaging	Polymorphism	2972; 3227; 4016
**Other genes**													
*CDH1*	c.1849G > A	p.Ala617Thr	rs33935154	missense	Likely Benign	Conflicting interpretations of pathogenicity	15.13	Class C55	Polymorphism	Tolerated	Benign	Disease Causing	3664; 4145; 4166
*TP53*	c.1010G > A	p.Arg337His	rs121912664	missense	Likely Pathogenic	Pathogenic	22.6	Class C25	–	Damaging	Probably damaging	Disease Causing	2699; 3056; 3227; 3662; 4264
	c.818G > A	p.Arg273His	rs28934576	missense	Likely Pathogenic	Pathogenic/Likely pathogenic	24.0	Class C25	Pathogenic	Damaging	Probably damaging	Disease Causing	3227

**Fig. 1:**
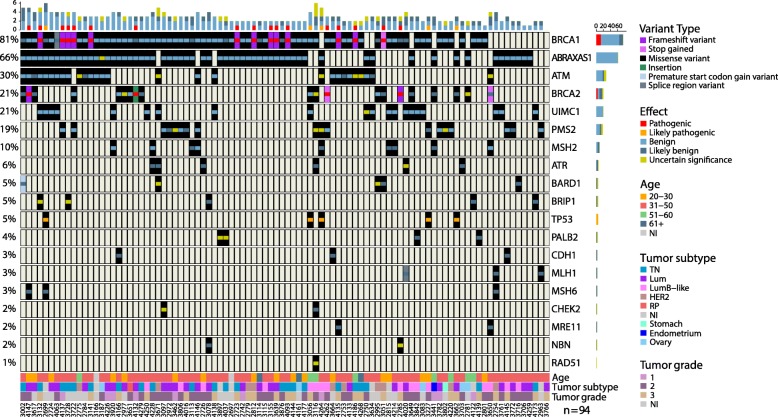
Molecular and clinical spectrum of prioritized variants found in 94 HBOC samples screened for variants in 21 DNA repair genes**.** The graph shows the frequency of prioritized variants identified per gene, and the effect of each variant according to VarSome. The samples were also classified according to the age at diagnosis, molecular subtype and tumor grade. In molecular subtype, TN = Triple-negative subtype; Lum = both Luminal A and Luminal B subtypes, when presenting positivity to estrogen and/or progesterone receptors and lack HER2 expression; LumHER = Luminal positive for all three markers; HER2 = when the HER2 protein is overexpressed with negative estrogen and progesterone receptors; PR = positivity to only progesterone receptors; NI = Not-informed. For the molecular subtypes we also indicate the cases that are not BC cases: Ovarian, Stomach and Endometrium. The bars and the numbers/scale on the top of the figure represent the type and number, respectively, of variants found per sample. The bars and the numbers/scale on the right side of the gene names represent the type and number, respectively, of variants found per gene. The numbers in the bottom represent the samples’ code

Figure 1 shows the most prevalent variants detected in the studied samples. About 11.2% (*n* = 9) were frameshift, stop gain, insertion or missense variants, previously described as pathogenic in *BRCA1, BRCA2* and *TP53* genes, with a prevalence of 23.4% (*n* = 22). The most prevalent pathogenic variant was the frameshift p.Gln1756Profs*74 (c.5266dupC) in *BRCA1* (ENSP00000350283.3) gene, present in half of the cases which exhibited *BRCA1* mutations (*n* = 11), followed by the variant p.Arg337His (c.1010G > A) in *TP53* (ENST00000269305.8), found in another 5 patients. Our results also introduce the first report of two known pathogenic variants in the Brazilian population: the p.Tyr3009Serfs*7 (c.9026_9030delATCAT) on *BRCA2*, and p.Arg273His (c.818G > A) in *TP53*.

In regard to *BRCA1* and *BRCA2* genes, we also identified five benign variants in the *BRCA1* gene presenting a high frequency in our HBOC cohort: the 3’UTR c.*421G > T, p.Pro871Leu (c.2612C > T), p.Glu1038Gly (c.3113A > G), p.Lys1183Arg (c.3548A > G), and p.Ser1613Gly (c.4900A > G). Based on previous results of our group which also found those variants in a high frequency in a small HBOC cohort (unpublished data), we sought to investigate whether those variants were segregating together and if they were associated with an increased HBOC risk. Haplotype analysis by Haplo.Stats program identified 5 haplotypes with frequencies above 1% (Table 3). Haplotype 2, with all five SNVs, was the second most frequent haplotype found (24.8%) in our study. However, this haplotype was significantly more frequent in the elderly cohort (*p* = 0.020), and was not associated with an increased HBOC risk.

**Table 3:** Haplotype estimation for five high frequency BRCA1 SNVs found in the HBOC cohort

Hp	p.Pro871Leu	p.Glu1038Gly	p.Lys1183Arg	p.Ser1613Gly	c.*421G > T	Hap.	Control	HBOC	*p*-value
	(CCG→CTG)	(GAA→GGA)	(AAA→AGA)	(AGT→GGT)	(G → T)	freq.	(*n = 136*)	(*n = 119*)	
1	Pro	Glu	Lys	Ser	G	0.546	0.533	0.563	0.532
2	Leu	Gly	Arg	Gly	T	0.248	0.292	0.199	**0.020**
3	Leu	Glu	Lys	Ser	G	0.136	0.129	0.143	0.633
4	Pro	Glu	Lys	Ser	T	0.028	0.017	0.038	0.172
5	Leu	Gly	Lys	Gly	T	0.028	0.017	0.038	0.172
6	Leu	Gly	Lys	Ser	T	0.008	0.007	0.007	NA
7	Leu	Gly	Lys	Gly	G	0.004	0.004	0.424	NA
8	Leu	Glu	Arg	Gly	G	0.003	0.003	0.000000002	NA
9	Leu	Gly	Arg	Gly	G	0.002	0.00000002	0.004	NA

*Hp* Estimated haplotypes, *Hap. freq.* General haplotype frequency found for all samples, *Control* Haplotype frequency found for the 136 elderly control samples, *HBOC* Haplotype frequency found for the 119 HBOC samples, *p-value* Haplotype score statistic p-value calculated by Haplo.stats, and considered significant when *p*>0.05 (in bold, the p-value considered as significant), *NA* When the haplotype score statistic *p*-value could not be calculated

To further investigate if there is any correlation between *BRCA1* haplotypes and HBOC risk, we performed the haplotype analysis using HBOC and control samples from another three cancer centers in Brazil: Porto Alegre Clinical Hospital (HPOA), A.C. Camargo Cancer Center (ACC) and Barretos Cancer Hospital (HCB). Haplotype analysis results were similar for all three centers. The Haplotype 2 (Table 3) were not significant in the other three centers (Haplotype in red, Additional file 2: Table S2), but Haplotype 3, which encompasses only the p.Pro871Leu SNV, showed a significant difference between HBOC and control groups in the three other cancer centers (*p* = 0.027; *p* = 0.007; *p* = 0.026 respectively) (Haplotype in bold, Additional file 2: Table S2), but also showed a higher frequency in the control group, suggesting no correlation with an increased risk of HBOC Syndrome. Once both variants and haplotypes were present in the elderly and other control samples, we suggest despite segregating together, those variants may merely constitute part of a polymorphic region and are not associated with hereditary cancer risk.

**Fig. 2:**
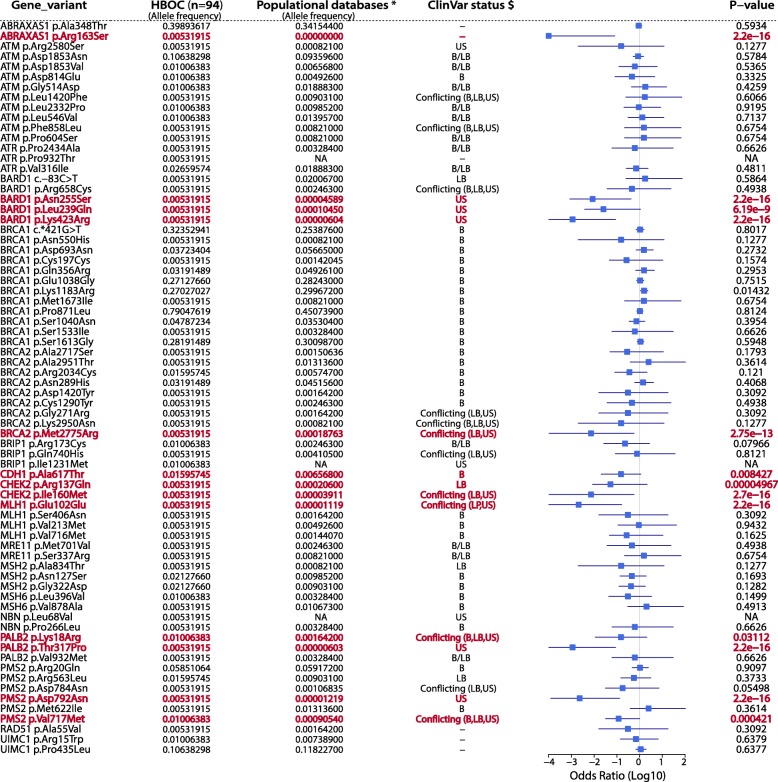
Association analysis of 72 prioritized variants with conflicting data on pathogenicity to HBOC risk. The risk association analyses were performed comparing the allele frequencies identified in our HBOC cohort to frequencies found in public databases (*) AbraOM, ExAC and 1000 Genomes. In ClinVar status ($), B = Benign; LB = Likely Benign; US = Uncertain Significance; P = Pathogenic; Conflicting = when presenting conflicting interpretations of pathogenicity. The association was made using Fisher’s exact test, and the p-values were assessed using the Pearson’s X^2^ test. The lack of allele frequencies in the databases made us unable to estimate the odds ratios (OR). The variants in red are those significantly associated with HBOC risk. NA = Not available (allele frequencies not reported by any populational database, or when was not possible to calculate the p-value due to the lack of allele frequency in the populational databases)

About 12.8% (*n* = 12) of the patients did not present any variants in the *BRCA1/BRCA2* genes (Fig. 1, and Additional file 1: Table S1). Most cases (76.6%) presented missense VUS or benign missense variants according to VarSome and ClinVar, which were qualified as being pathogenic by the in silico prediction tools, which may unable the clinical interpretation and risk estimation during the genetic counselling for carriers. The association study with these variants identified 8 genes carrying 13 variants as significantly associated with an increased risk to HBOC when compared to the allele frequencies described in public databases. Genes such as *BARD1*, *CHEK2*, *PALB2* and *PMS2* presented more than one variant associated with risk (Fig. 2).

**Fig. 3:**
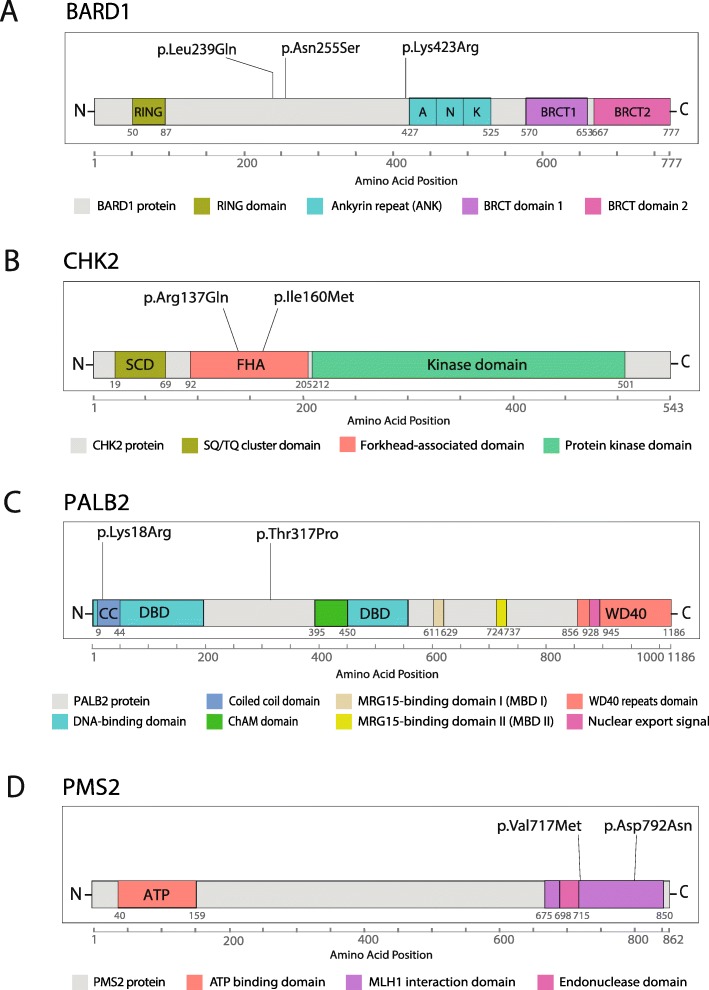
Schematic representation of BARD1, CHK2, PALB2 and PMS2 proteins and the variants associated with increased risk to HBOC. **a** Linear representation of BARD1 protein depicting the RING, Ankyrin (ANK), and BRCT domain boundaries [46], and the three variants found in that gene; (**b**) CHK2 depicting the SQ/TQ cluster domain (SCD), forkhead-associated domain (FHA), and the kinase domain (KD) [47], showing the localization of the two variants identified in that gene; (**c**) PALB2 protein with its main domains depicted: coiled coil, ChAM, MRG15-binding domain I and II (MBD I and II), WD40 repeats domain, and the nuclear export signal (NES) [48], showing the variants found as significantly associated HBOC risk; and (**d**) PMS2 with its ATP and MLH1 binding domains, and its endonuclease domain [49], depicting the variants identified in that gene. The graphs were built using the lolliplot function of the GenVisR package, on R environment (RStudio, version 1.2.1335), and were adapted by the authors

The prevalence of variants associated with HBOC was about 16% (*n* = 15), and most of them (*n* = 13) were present in double heterozygosis variants with conflicting data on pathogenicity in *BRCA1/BRCA2*. *BARD1, CHEK2*, *PALB2* and *PMS2* presented more than one variant associated with risk (Fig. 3), and the variant p.Ala617Thr (c.1849G > A) in *CDH1* gene presented the highest allele frequency (AF = 0.01595745). One patient presented a pathogenic variant in *BRCA1* in double heterozygosity with one *BARD1* prioritized variant (Fig. 1, and Table 2).

All patients carrying variants associated with an increased risk, as well those who did not present any *BRCA1/BRCA2* variants tested negative for *BRCA1/BRCA2* CNVs.

**Fig. 4:**
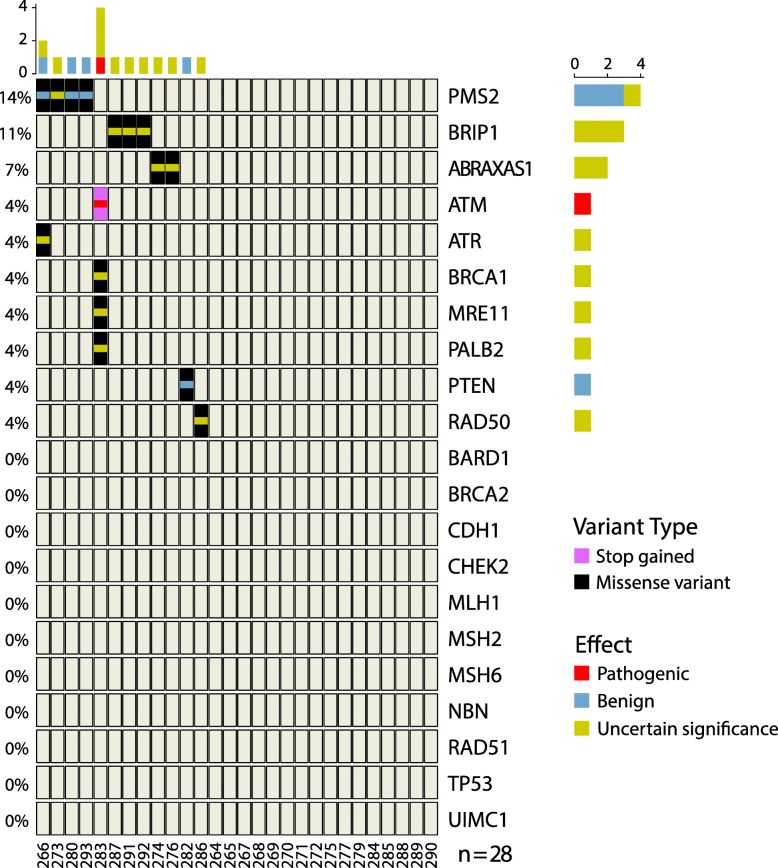
Spectrum of variants found in 21 DNA repair genes screened in 28 samples of an elderly cohort from Southeast Brazil. The heatmap shows the frequency of missense and stop gain variants found per gene, and the effect of each variant according to VarSome

As expected, in the elderly cohort we identified only a small number of coding variants classified as pathogenic or of uncertain significance (VarSome and ClinVar), when looking at the 21 genes screened in our HBOC cohort (Fig. 4). However, none of the variants described in the HBOC patients were found in the elderly samples used as control. Despite the small sample size available for the elderly cohort, our data confirms that cohort constitute a proper control in hereditary cancer studies.

### Clinical characteristics of germline variants-carriers

The prevalence of pathogenic variants in *BRCA1* and *BRCA2* was about 18% (*n* = 17), with only four patients presenting *BRCA2* pathogenic variants. We observed that 90% of carriers of *BRCA1* pathogenic variants presented with high grade tumors (grade 3) while about 80% of *BRCA2* carriers presented with tumors with grades I and II. Additionally, most of BRCA1-variant carriers were diagnosed with triple negative BC (Fig. 1). The non-*BRCA1/BRCA2* group also presented high frequency of intermediate to high grades tumors (grades 2 and 3) (Fig. 1, Table 1), which may suggest that other genes are associated with moderately-poorly differentiated tumors as is known for *BRCA1*/*BRCA2*-carriers [50]. The presence of metastasis was strongly correlated with death (*p* = 7.85e-12) since 13 out of 14 patients that died presented distant metastasis. We did not find any association between tumor clinical staging and the genotypes.

**Fig. 5:**
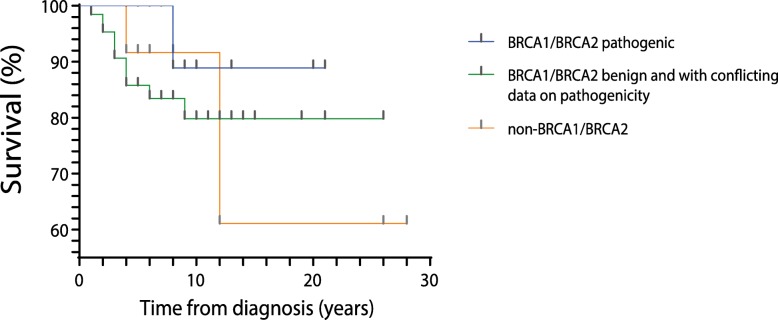
Survival of patients after clinical diagnosis according to the genotype regarding the presence of *BRCA1/BRCA2* variants**.** The small grey bars represent the censured data (when despite continuous monitoring of outcome event, the death does not occur within the study duration), and the time of follow-up after clinical diagnosis, since we studied patients diagnosed with cancer 28 years ago and some diagnosed 4 years ago. Conflicting data on pathogenicity refers to VUS and benign variants that were predicted as pathogenic by the in silico tools. *BRCA1/BRCA2* pathogenic *n* = 17, *BRCA1/BRCA2* benign and with conflicting data on pathogenicity *n* = 65, non-*BRCA1/BRCA2 n* = 12. We did not find any significant difference between the genotypes (Logrank test for trend, *p* = 0.3439)

A total of 12 individuals (12.8%) did not present any variants or CNVs in *BRCA1/BRCA2* and were grouped as non-*BRCA1/BRCA2* patients. This group presented variants in *ABRAXAS1, ATM, ATR, BARD1, CDH1, MLH1, MSH6, PMS2, TP53* and *UIMC1* genes. All non-*BRCA1/BRCA2* patients were BC cases, showing a median age at diagnosis of 36.5 years and a median survival of 8 years (Table 1). However, we did not observe any association with death with the genotype of the patients. Surprisingly, the patients that presented pathogenic variants in *BRCA1/BRCA2* showed a trend towards better survival with most of cases that died being the ones that presented VUS, benign or no variants in *BRCA1/BRCA2* genes (Fig. 5).

## Discussion

Genes such as *BRCA1, BRCA2* and *TP53* presented pathogenic variants in 23.4% (*n* = 22) of the investigated cases. The only study with a multi-gene analysis in Brazil has shown genes such as *BRCA1, BRCA2, ATM, ATR, MLH1, MSH2* and *MSH6* carrying pathogenic variants but with a much lower frequency (9.5%) [24].

The most prevalent variant was the frameshift p.Gln1756Profs*74 (c.5266dupC) in *BRCA1*, identified in 11.7% of patients. This variant was also described in the study of Timoteo et al. (2018) [24], but with a frequency of only 3%. This variant is commonly found in South American populations, being well described in Brazil, especially in ovarian cancer cases [51, 52], although it was found only in breast cancer cases in our HBOC cohort. It is a founder Ashkenazi Jewish variant and it is very common among North European populations [53]. This may explain the high frequency found in the Southeast of Brazil, which is marked by a strong European ancestry [54].

Four patients presented the following variants in *BRCA2* genes: p.Ala938Profs*21; p.Tyr3009Serfs*7; p.Arg3128Ter and, the third most common variant within Brazilian population, the c.156_157insAlu. The Alu retroelements are fragments of approximately 300 nucleotides that are reported as being inserted in many genes such as *BRCA1* and *BRCA2* and are related to an increased cancer risk [55, 56]. The Alu insertion in *BRCA2* exon 3 was first reported by Teugels et al. (2005) [57] as a Portuguese founder variant in HBOC patients, and due to the Portuguese immigration during the Brazilian colonization, this variant is frequently found in Brazilian populations [55]. The pathogenicity of this insertion is attributed to the exon 3 skipping, which causes the loss of the PALB2 and RAD51 binding region, essential to homologous recombination repair [48].

Five patients also presented the pathogenic variant p.Arg337His in *TP53* gene. This is a founder variant of South Brazil, known as segregating in families with sarcomas, adrenocortical and choroid plexus carcinomas, and breast cancer at early onset [30, 58]. It is located in the oligomerization domain of p53 and as well as the segregation studies, it has been shown that this variant is associated with a decreased oligomerization and transcriptional activities of p53 [59, 60].

However about 76.6% of the cases presented VUS and variants with conflicting data on pathogenicity in *BRCA1/BRCA2* as well as in other investigated genes based on data from VarSome, ClinVar or pathogenicity tools herein employed. In this group we found one patient carrying the previously undescribed variant p.Pro932Thr (c.2794C > A) in *ATR* gene, which is predicted as pathogenic/possibly pathogenic by all in silico tools used in this study. This patient also presented variants in other genes such as *BRCA1, UIMC1* and *MLH1*, but tested negative for *BRCA1/BRCA2* CNVs. It is a case of unilateral BC with lymph node metastasis diagnosed at 40 years old and with a 4-year survival after diagnosis.

For those cases who did not present any pathogenic variant we observed a high frequency of the five *BRCA1* benign variants: the 3’UTR c.*421G > T, p.Pro871Leu (c.2612C > T), p.Glu1038Gly (c.3113A > G), p.Lys1183Arg (c.3548A > G) and p.Ser1613Gly (c.4900A > G). As shown in Table 3, these variants were segregating together, and constituted the second most frequent haplotype found in this study. Despite this, the haplotype containing the five SNVs was significantly more frequent in elderly cohort (29.2%) when compared to HBOC cases (19.9%) (*p* = 0.020), which suggests that these variants are not associated with an increased risk to HBOC. Indeed, four of these variants were previously described as presenting a high frequency in a healthy cohort in an ethnic dependent manner, with p.Pro871Leu presenting high African and European ancestry, and p.Glu1038Gly, p.Lys1183Arg, and p.Ser1613Gly, associated with the Central Asiatic ethnic component [61]. It may explain the high frequency of these variants in the studied population.

The genes *ABRAXAS1*, *UIMC1* and *ATM* also presented a high frequency of missense variants in our HBOC cohort. About 66% of the patients carry the variant p.Ala348Thr (c.1042G > A) in *ABRAXAS1*, which is not characterized by ClinVar but is predicted as pathogenic by 3 in silico tools. The allele frequency for this variant was 0.4 in our cohort, and population databases describe p.Ala348Thr with a MAF = 0.34 in Brazil [42] and MAF = 0.42 worldwide [62], which corroborates the ACMG/AMP classification of p.Ala348Thr as a benign variant. The p.Pro435Leu (c.1304C > T) in *UIMC1* is another VUS not described on ClinVar that presented a high allele frequency (0.10) in our HBOC cases. It also has a high MAF in the population databases (0.12 [42] and 0.24 [62]). Together with Abraxas, RAP80 is part of the BRCA1-A complex which is important for recruiting BRCA1 to double-strand break (DSB) sites [63] and studies have shown that truncating variants in both proteins are associated with increased irradiation sensitivity, deficient BRCA1 recruitment to DSB sites and genomic instability [64–67]. Three patients that carried only these two variants were evaluated for *BRCA1/BRCA2* CNVs and all tested negative. Due to their high allele frequency, these variants are classified as benign by the ACMG/AMP, however, a more accurate characterization is mandatory to address a clinical significance for these variants, since both are not characterized yet and we cannot discard its contribution to risk following a polygenic inheritance pattern, for example.

Another gene that presented high frequency of variants was *ATM* (Fig. 1). About 16.8% out of the patients that presented variants in *ATM* carried the variant p.Asp1853Asn (c.5557G > A), characterized as benign by ClinVar and VarSome. Studies with this variant have shown that it is not associated with an increased risk to HBOC [68].

We also observed a high frequency of missense variants in MMR genes, especially for *PMS2* and *MSH2* which were mutated in 19 and 10% of the cases, respectively (Fig. 1). Despite truncating variants in those genes being the cause of Lynch Syndrome (LS), it is common to find an overlap between HBOC and LS cases since both syndromes are well known for predisposition to BC and OC [69]. Many studies have reported MMR genes as being associated with an increased risk to HBOC [70–72] and indeed, they have been taken into account by NCCN guidelines for the clinical management of patients at risk of hereditary BC and OC [4, 73].

However, most patients (76.6%) carry missense VUS or variants presenting conflicting data on pathogenicity. The association analysis based on Brazilian [42] and worldwide public databases [62] revealed 13 variants in *ABRAXAS1, BARD1, CDH1, CHEK2, MLH1, PALB2* and *PMS2* genes associated with HBOC, with a prevalence of 15.9% (Fig. 2). The variant p.Ala617Thr (c.1849G > A) in *CDH1* gene was the most frequent among the studied cases. Differently to the other genes, *CDH1* encodes the adhesion protein E-cadherin and variants in this gene are associated with defects in cell adhesion, an increase in the invasive activity and, consequently, metastasis [74]. *CDH1* truncating variants are associated with risk to gastric diffuse cancer and in fact, one patient presented familial history of gastric cancer, however, all three cases presented BC or fulfilled NCCN criteria for HBOC risk. This variant has been previously described in the Brazilian population as pathogenic [24, 75] but functional assays with cells expressing the mutated protein have shown wild type morphology and normal proliferation and migration activities [76], which suggests this variant may not lead to protein truncation.

The *BARD1* was the gene that presented more variants associated with HBOC risk. BARD1 form heterodimers with BRCA1 playing an important role as both E3 ubiquitin ligase as homologous repair mediators by recruiting RAD51 to DSB sites [77].

Variants in this genes have been associated with a deficiency in HR and increased sensitivity to DNA damage, classifying *BARD1* as a gene of moderate penetrance to BC and OC [23, 77–79]. All three associated variants are described as VUS on ClinVar, but p.Asn255Ser (c.764A > G) and p.Lys423Arg (c.1268A > G) lack studies characterizing their effects on protein functions. Indeed, this is the first study reporting both variants in a HBOC cohort from Brazil. The third variant p.Leu239Gln (c.716 T > A) has been described in the North American population and was also characterized as a VUS [80]. Despite being predicted as likely benign by VarSome, p.Leu239Gln and p.Asn255Ser are predicted as pathogenic by 2 out of 6 in silico tools and are located between the RING and ANK BARD1 domains (Fig. 3a). RING is the region of BRCA1 binding and it is important for heterodimers formation [81]. p.Leu239Gln was found in double heterozygosis with the pathogenic variant p.Trp1836Ter in *BRCA1*, but p.Asn255Ser was identified in a non-*BRCA1/BRCA2* BC patient. Regarding p.Lys423Arg variant, it is located in ANK domain which plays an important role in apoptosis activation due to p53 binding [82]. Despite ANK not being related to the DNA repair process, the evaluation of variants located between amino acids 460–560 have shown an HR deficiency demonstrating that this domain is also important to a correct DNA repair [77]. In fact, three in silico tools classified this variant as pathogenic, however, only functional or segregation analyses are required to confirm the suggested pathogenic effect of those variants.

The role of *BRCA1/BRCA2* genes in the HBOC pathogenesis is already well characterized. The VUS p.Met2775Arg (c.8324 T > G) in *BRCA2* was identified in one BC patient in double heterozygosis with other associated variants such as p.Arg137Gln in *CHEK2* and p.Val717Met in *PMS2*. p.Met2775Arg has been described in prostate cancer cases and is characterized as possibly pathogenic by 4 in silico prediction tools despite this variant not affecting conversed residue [83, 84]. It is located in the C-terminal of BRCA2 proteins, which is important for single strand DNA binding as well as for delivering RAD51 molecules to DSB sites, allowing for a correct homologous recombination repair [85]. It indicated that the integrity of this region is essential for a correct HR. Taking into account that this patient presented three other variants significantly associated with HBOC, we suggest this genotype may have an additive effect on breast cancer risk in this case.

*CHEK2* gene also presented two variants associated with risk (Fig. 3b). Chk2 plays an important role in signalling the DNA damage through phosphorylating effector proteins such as BRCA1 [86]. Both variants p.Arg137Gln and p.Ile160Met are located in the FHA domain (Fig. 3b), which after Chk2 phosphorylation and KD domain activation, binds to SCD domains of other Chk2 activated protein, forming dimers that convert into active monomers, signalling the DNA damage [87]. p.Arg137Gln and p.Ile160Met are predicted as pathogenic/possibly pathogenic by two and four in silico tools, respectively. However, functional analyses have shown that p.Arg137Gln is not associated with protein instability and HR deficiency [88–90] which corroborates with its probable benign classification by VarSome and ClinVar. On the other hand, p.Ile160Met is a VUS that has been related to a moderate HR deficiency [91], and in fact, carriers of p.Ile160Met variant presented a worse clinical condition, presenting bilateral BC and death after pulmonary, bone and hepatic metastases in this study. Due to the localization and the clinical features, we suggest that p.Ile160Met may play a role in the risk of HBOC.

Besides presenting the most frequent variant found in this HBOC cohort, *ABRAXAS1* also presented the p.Arg163Ser (c.489G > T) variant as being significantly associated with HBOC relative risk (Fig. 2). It is a VUS according to VarSome, which is not described by ClinVar but is characterized as pathogenic by 5 out of 6 prediction tools. p.Arg163Ser is located in the Pad1 domain in the N-terminal region of ABRAXAS, an important RAP80 and other signalling proteins binding domain [92]. Both proteins are mandatory for BRCA1 recruitment to DSB sites and variants affecting that region of ABRAXAS may affect the correct DSBs signalling [64, 93].

The synonymous variant p.Glu102Glu (c.306G > A) in *MLH1* is predicted as likely benign by VarSome, and is characterized as VUS by ClinVar but was associated with HBOC risk (Fig. 2). It affects a splicing region in the end of *MLH1* exon 3. Due to this, p.Glu102Glu is predicted as pathogenic by all in silico tools that return pathogenicity scores for synonymous variants (CADD, UMD predictor and mutation taster). This variant is also described in BC samples of TCGA. Although the publicly available data on TCGA comprises solely somatic variants, it may corroborate the association with increased HBOC risk. The patient carrying this variant was a BC case who also presented other benign variants in *MLH1* and *BRCA1*, a VUS in *UIMC1*, as well as the novel variant p.Pro932Thr in *ATR.* As previously described, truncating variants on MMR proteins are known for increasing the risk for both BC and OC [70–72]. However, there is no further evidences of the deleteriousness of this variant.

Regarding *PALB2* gene, two N-terminal variants were found to be associated with HBOC risk. Despite *PALB2* biallelic mutations being associated with Fanconi Anemia, heterozygous variants are known to confer a moderate risk to BC [48, 94]. According to VarSome, p.Arg18Lys (c.53A > G) is a VUS which also presents conflicting interpretations of pathogenicity by ClinVar, and is predicted as pathogenic by 3 in silico tools. It is located in the PALB2 coiled coil domain (Fig. 3c), the BRCA1 binding region, but studies have shown that this variant does not affect the PALB2-BRCA1 interaction although it promotes a reduction on HR activity [95]. This variant was found in two BC patients, with one case being a triple-negative subtype (TNBC) (Table 2, and Additional file 1: Table S1). The p.Thr317Pro (c.949A > C) is a VUS identified in a TNBC case which presented lymph nodes metastasis. It is located near the DBD domain, which important for PALB2 DNA binding [48] (Fig. 3c), but differently to p.Arg18Lys, there is no report of this variant in other studies, and it is characterized as possibly pathogenic by two prediction tools. Recently, a study encompassing the functional characterization of 44 *PALB2* missense variants evidenced that both variants are not affecting the evaluated PALB2 protein functions [96]. 

The last risk-associated gene was *PMS2*, which presented two C-terminal variants located in the MutL domain that together with the N-terminal region constitute the MLH1 binding region (Fig. 3d). This region is important for MutLα heterodimers formation, necessary for the correct mismatched DNA fragment excision [97]. The p.Val717Met (c.2149G > A) is a VUS that presents conflicting information of pathogenicity by ClinVar database and only AlignGVGD does not predict it as pathogenic. Functional assays have demonstrated a protein stability and MMR proficiency, however, the samples carrying this variant presented microsatellite instability [98]. The p.Asp792Asn (c.2374G > A) variant was identified in a gastric diffuse cancer patient, the only man in our cohort, which ended in death 3 years after the diagnosis. It has been described as presenting a moderate decrease in mismatch repair activity [99], which corroborates with our analysis association. Due to this, we suggest that these variants may be related to increased risk to HBOC, but segregation studies and functional characterization are mandatory to access the contribution of these variants to HBOC etiology.

## Conclusions

Our study is comprised of the third multi-gene screening in HBOC patients in the Brazilian population, showing a higher frequency of pathogenic variants than previously reported [24]. In addition, our work expands the landscape of variants linked to HBOC syndrome in the Brazilian population, and also depicts the first report of the novel *ATR* missense variant p.Pro932Thr (c.2794C > A). This study also presents a descriptive characterization of variants found in HBOC patients, evidencing about 16% of patients carrying variants significantly associated with HBOC risk, and constitutes the first report of missense variants on *ABRAXAS1, BARD1, BRCA2, CHEK2, PALB2* and *PMS2* in Brazil. As well as segregation analyses and functional characterization, which are mandatory to confirm the deleteriousness of the variants described here, these results bring insights to the contribution of other genes to HBOC pathogenesis. Our data also aggregates epidemiologic information about the prevalence of germline variants in DNA repair genes in the Brazilian population, which together with further characterization will help guide the clinical decision and risk assessment for patients at increased risk to HBOC in the future.

## Supplementary information

**Additional file 1: Table S1.** Clinical characterization of the HBOC cohort. NI = Not-informed; CNS = Central Nervous System. The tumor size, lymph node staging and the metastasis status are reported according to MOC Brazil guidelines for tumor staging (https://mocbrasil.com/).

**Additional file 2: Table S2.** Haplotype estimation for five high frequency BRCA1 SNVs in three different Cancer Centers in Brazil**.** Here we show only haplotypes with frequencies higher than 1%. In red, the haplotype identified as significantly more frequent in the elderly cohort, in our HBOC cohort analysis. In bold, the haplotype that was significantly more frequent in the control group of all three other Brazilian Cancer Centers. HPOA = Hospital das Clínicas de Porto Alegre, Porto Alegre, RS, Brazil; ACC = A.C. Camargo Cancer Center, São Paulo, SP, Brazil; HCB = Barretos Cancer Hospital, Barretos, SP, Brazil. Hp = estimated haplotypes. Hap. freq. = haplotype frequency. *p*-value = haplotype score statistic p-value calculated by Haplo.stats. NA = when the haplotype score statistic *p*-value could not be calculated.

## Data Availability

The publicly available datasets analyzed during the current study are available in the AbraOM [42], 1000 genomes [43] and ExAC [44] databases. The authors declare that all relevant data are included in the article and its additional material files, and that it is also available from the corresponding author by request. The WES data of the elderly cohort supporting some analysis performed in this article is available in the Brazilian Initiative on Precision Medicine Project (BIPMed; http://bipmed.org).

## Abbreviations

ACMG/AMP: American College of Medical Genetics and Genomics and the Association for Molecular Pathology
ASCO: College of American Pathologists
BC: Breast Cancer
DSB: Double Strand Breaks
ER: Estrogen Receptors
HBOC: Hereditary Breast and Ovarian Cancer Syndrome
HER2: human epidermal growth factor receptor 2
IDC: Invasive Ductal Carcinoma
indels: insertion/deletions
IR: Irradiation
NCCN: National Comprehensive Cancer Network
NGS: Next Generation Sequencing
OC: Ovarian Cancer
PR: Progesterone Receptors
SNP: Single Nucleotide Polymorphisms
SNVs: Single Nucleotide Variants
TCGA: The Cancer Genome Atlas
TN: Triple-negative
VUS: Variants of Unknown Significance
